# Caregiver perceptions and utilization of oral rehydration solution and other treatments for diarrhea among young children in Burkina Faso

**DOI:** 10.7189/jogh.06.020407

**Published:** 2016-12

**Authors:** Peder Digre, Evan Simpson, Shannon Cali, Belinda Lartey, Melissa Moodley, Ndack Diop

**Affiliations:** 1 PATH, Seattle, WA, USA; 2 Linksbridge, Seattle, WA, USA; 3 Ipsos Healthcare, London, UK; 4 PATH, Dakar, Sénégal

## Abstract

**Background:**

More than 500 000 young children die from dehydration caused by severe diarrhea each year, globally. Although routine use of oral rehydration solution (ORS) could prevent almost all of these deaths, ORS utilization remains low in many low–income countries. Previous research has suggested that misperceptions among caregivers may be an obstacle to wider use of ORS.

**Methods:**

To better understand the extent of ORS utilization and the reasons for use or non–use in low–resource settings, the project team conducted a semi–structured, quantitative survey of 400 caregivers in Burkina Faso in 2014. All caregivers had a child below the age of five who had diarrhea lasting 2 days or more in the previous 2 months.

**Results:**

Although more than 80% of caregivers were aware of ORS, less than half reported using it to treat their child’s diarrhea. Replacing fluids lost due to diarrhea was considered a low priority by most caregivers, and many said they considered antibiotics more effective for treating diarrhea. Users and non–users of ORS held substantially different perceptions of the product, though all caregivers tended to follow recommendations of health care workers. A significant proportion of users reported difficulty in getting a child to drink ORS. Costs and access to ORS were not found to be significant barriers to use.

**Conclusions:**

Misperceptions among caregivers and health workers contribute to low utilization of ORS. Better caregiver understanding of diarrheal disease and the importance of rehydration, as well as increased recommendation by health workers, will help to increase ORS utilization. Improving product presentation and taste will also help to increase use.

Among children under five years of age, diarrhea remains a significant cause of morbidity and mortality. It is estimated that more than 500 000 children in this age group die each year from dehydration caused by diarrhea, globally. In Burkina Faso alone, more than 6000 deaths of children under five years of age were due to diarrhea in 2013. [1] This is despite the availability of an inexpensive and highly efficacious treatment: oral rehydration solution (ORS). ORS was developed in the 1970s, and its use is credited with saving millions of lives. It is estimated that more than 90% of all diarrhea deaths could potentially be avoided with universal coverage of ORS [2]. Despite the benefits of ORS, and the widespread knowledge of ORS by mothers and caregivers, coverage has remained low in many developing countries and regions. In Burkina Faso in 2010, more than 75% of caregivers reported having heard of ORS, but only fewer than 25% reported using ORS [3].

The reasons behind this “know–do” gap have been the source of widespread speculation. One thought about why caregivers do not use ORS is that it fails to meet expectations for treating the symptoms and does not stop the diarrhea [4]. Other reasons for nonuse include the bad taste and the fact that it lacks the appearance of a “real” medicine [5]. Low coverage of ORS has also been attributed to the availability of alternative products such as herbal remedies and antibiotics.

Understanding the full extent of ORS utilization and the reasons for use or nonuse is important for governments, nongovernmental organizations (NGOs), and public health programs that are developing interventions to increase coverage. It is also important to understand the extent of use of alternative interventions, such as antibiotics, and the reasons for use.

From 2010 to 2014, PATH and colleagues at Ipsos Healthcare conducted extensive quantitative and qualitative surveys of caregivers and formal and informal health care providers in Burkina Faso, India, Kenya, Nigeria, and Zambia to probe the reasons for use or nonuse of ORS and other interventions to manage diarrhea [6]. This paper presents the findings from a quantitative survey of caregivers in Burkina Faso that examined the use of various interventions, the rationale for treatment decisions, treatment costs, and expectations associated with their experience in treating diarrhea in the past two months in a child under five.

## METHODS

The project team conducted a quantitative survey of 400 caregivers in Burkina Faso in June and July 2014. A complementary quantitative survey of 250 pharmacy staff and health care workers (“providers”) was simultaneously conducted but is not covered in this report. Development of the survey instruments was informed by a formative qualitative research process, which involved 60–minute, face–to–face, in–depth interviews with caregivers (predominantly mothers) who were aware of ORS.

### Selection and description of participants

All surveyed caregivers had a child between six months and five years of age who had an episode of diarrhea that occurred less than two months prior to the interview and that lasted for more than two days. **Table 1** presents a demographic profile of caregivers. We used quota sampling methods to identify appropriate numbers of urban and rural respondents

**Table 1:** Demographics of surveyed caregivers (base: all caregivers, n = 400)*

Variable	Percentage (%)
**Age of caregiver:**	
18–20	7
21–24	21
25–34	51
35–44	20
45–54	2
**Number of children aged between 6 months and 5 years per caregiver:**	
1	73
2	25
3+	2
**Age of child:**	
At least 6 months old but under 1 year	24
At least 1 year old but under 2 years	37
At least 2 years old but under 3 years	26
At least 3 years old but under 5 years	31
**Socioeconomic class:**†	
C1	1
C2	10
D/E	88
**Setting:**	
Rural	62
Urban	38
**Religion:**	
Christian	30
Muslim	66
Other	4
**Primary language:**	
French	22
Moore	36
Dioula	28
Other	14
**Location:**	
Centre	23
Boucle du Mouhoun	21
Hauts-Bassins	22
Nord	18
Est	17

*Selection criteria: caregivers with a child under 5 who had diarrhea in the last 2 months lasting 2 days or more.

† Socioeconomic levels: A is highest and E lowest. Socioeconomic classification was based on the standard systems used for commercial market research in the respective countries; in Burkina Faso, as described in the Oracle General Consumer Survey – Brand Values Segmentation (GCS-S) data collection tool.

The survey covered five regions – Centre (including Ouagadougou, the capital), Boucle du Mouhoun, Hauts–Bassins, Nord, and Est – representing the country’s major socio–cultural groups (Table S1 in **Online Supplementary Document(Online Supplementary Document)** for demographics of each region). We also used quotas for regions, with the sample distributed according to the relative population of each region. Within regions, sampling points were purposively selected, and interviewers used systematic random sampling to identify respondents during recruitment (Table S2 in **Online Supplementary Document(Online Supplementary Document)**). Unlike the qualitative research, the quantitative research did not set criteria related to ORS awareness or experience so we could establish a representative measure of population awareness and usage of ORS and other diarrhea treatments. More information about the selection of participants can be found in Appendix S2 in **Online Supplementary Document(Online Supplementary Document)**.

### Survey focus and design

The survey focused on the child’s most recent episode of diarrhea. In the 60–minute interview, topics probed included diarrhea duration, treatments used, sequence/timeframe of administration, and caregivers’ expectations for each treatment (eg, “what did you think [the treatment] would do for your child?”). Caregivers were also asked about the treatment source, spending on treatment, care–seeking behaviors, and dosing of ORS and homemade sugar–salt solution (HSSS). The survey did not probe related costs such as transport or lost work time. Peak dosing estimates were calculated using conservative assumptions to err on the side of overestimating the amounts given. The survey questionnaire is available upon request from the corresponding author.

Other topics covered included awareness and previous use of treatments (ORS sachets, HSSS, other home remedies, herbal remedies, antibiotics, anti–motility drugs, and zinc syrups/tablets). After recording spontaneous recall of treatments used, interviewers used localized illustration cards to prompt or to assist in recall of treatment types. They also assessed perceptions of ORS by using positive–negative statement pairs and evaluated willingness to pay for a diarrhea treatment. Attribute association was carried out based on the four main treatments used (established previously in qualitative research): ORS, antibiotics, anti–motility drugs, and HSSS. This involved caregivers selecting which treatments they felt fulfilled each attribute (such as “easy for children to take,” “stops the diarrhea,” and “not expensive”). Interviewers also asked caregivers to rank the four treatments on effectiveness and value (HSSS was not included for the perception of value).

The survey was pretested with a small number of caregivers (n = 20) in Ouagadougou. This ensured that survey questions were appropriate and refined before widespread data collection.

### Analysis

Quantitative data were analyzed for all respondents as a whole as well as for key groups, such as ORS users vs ORS non–users (based on usage at last episode of diarrhea) and urban vs rural respondents. We also evaluated data according to demographic and regional splits.

Data from open–ended questions were analyzed through a similar procedure. The process began with review of verbatim responses for each question. Key common themes were identified for each question, as well as factors associated with each theme. This represented a code frame. Each verbatim response was then analyzed and assigned to its appropriate code.

## RESULTS

### Duration of illness, treatments used and ORS dosing

Caregivers reported that the average diarrhea episode lasted 4 days (SD ± 1.96 days), with 15% of cases lasting 6 to 10 days. The vast majority of caregivers (84%) took some form of action when they realized their child had diarrhea. Notably, a greater number of caregivers in the Est region decided to “wait and see” before acting (42% vs 16% overall). Among caregivers who reported taking action after realizing their child had diarrhea (n = 385), 46% said they gave their child a special kind of food that would make him or her feel better or adjusted the child’s diet in some way. Among these caregivers, 33% reported that they stopped or avoided certain food types, and 27% said they increased liquid given to the child.

When asked about treatments, 328 caregivers (82%) said they were aware of ORS as a treatment option, and 385 (96%) administered some form of treatment. Among those who administered treatment during the last episode, less than half reported using ORS, even when illustration cards were used to prompt responses (**Table 2**). However, this is twice the coverage reported in a meta–analysis by Wilson et al. in 2012 [7]. Antibiotics were the second most popular primary treatment, followed by zinc syrup or tablets. Among caregivers who used more than one treatment (n = 166; 43% of those using treatment), there were many secondary treatments: 33% used zinc remedies (19% syrup; 14% tablets), 20% used antibiotics, 15% used anti–motilities, 14% used herbal remedies, and 10% used ORS (**Table 3**).

**Table 2:** Usage at last episode*

	Spontaneous response: without illustration cards						Prompted usage: with illustration cards					
All respondents using a treatment at last episode (n = 385)						All respondents using a treatment at last episode (n = 385)					
Total (n = 385)	Centre (n = 87)	Boucle du Mouhoun (n = 81)	Hauts-Bassins (n = 80)	Nord (n = 70)	Est (n = 67)	Total (n = 385)	Centre (n = 66)	Boucle du Mouhoun (n = 34)	Hauts-Bassins (n = 35)	Nord (n = 38)	Est (n = 35)
	A	B	C	D	E		A	B	C	D	E
**All treatments administered**	%	%	%	%	%	%	%	%	%	%	%	%
ORS	44	22 (B, C, D, E)	56	55	41	46	46	24 (B,C,D,E)	57	56	46	49
Antibiotics	34	53 (B,C,E)	16	20	50	34	37	55 (B,C)	16	20	57 (B, C, E)	40 (B, C)
Zinc syrup	19	5	15 (A,D)	45 (A, B, D, E)	4	25 (A,D)	20	6	16 (A)	44 (A, B, D)	6	31 (A, B, D)
Herbal remedies	15	13	27	15	9	7	16	13	28 (A, D, E)	16	11	10
Zinc tablets	8	1	4 (A, B)	18 (A)	9 (A)	12	11	1	4	22 (A, B)	14 (A, B)	15 (A, B)
Anti-motilities	7	18 (B, C, D, E)	6 (D)	4	0	4	10	24 (B, C, D, E)	9 (D)	10 (D)	1	4
Homemade sugar and salt solution (HSSS)	4	3	5	1	0	10 (C, D)	5	3	7 (D)	2	0	13 (A, C, D)
Other home remedies (other than HSSS)	2	1	5	0	0	1	4	3	9 (D)	2	0	3
Antibiotic injections	0	0	0	0	0	0	1	0	0	1	1	1
**Prompted usage: with illustration cards**												
	ORS users (n = 177)						ORS non-users (n = 208)					
	Total (n = 177)	Centre (n = 87)	Boucle du Mouhoun (n = 81)	Hauts-Bassins (n = 80)	Nord (n = 70)	Est (n = 67)	Total (n = 208)	Centre (n = 66)	Boucle du Mouhoun (n = 34)	Hauts-Bassins (n = 35)	Nord (n = 38)	Est (n = 35)
		A	B	B	D	E		A	B	C	D	E
**All treatments administered**	%	%	%	%	%	%	%	%	%	%	%	%
ORS	100	100	100	100	100	100	0	0	0	0	0	0
Antibiotics	18	19	15	4	31 (C)	27 (C)	54	67 (B, C)	18	40 (B)	79 (B, C, E)	51 (B)
Zinc syrup	20	0	20 (D)	36 (D)	0	30 (D)	21	8	12	54 (A, B, D)	11	31 (A, D)
Herbal remedies	10	5	13	7	16	6	21	15	47 (A, D, E)	29 (D)	8	14
Zinc tablets	14	0	2	31 (A, B)	19 (A, B)	15 (A, B)	8	2	6	11	11	17
Anti-motilities	3	14	4	0	3	0	16	27 (D, E)	14 (D)	23 (D)	0	9
Homemade sugar and salt solution (HSSS)	8	10 (D)	13	4	0	15 (D)	2	2	0	0	0	11 (A, B, C, D)
Other home remedies (other than HSSS)	5	10	9	4	0	3	2	2	9	0	0	3
Antibiotic injections	2	0	0	2	3	3	0	0	0	0	0	0

ORS – oral rehydration solutions

*Letters (A, B, C, D, E) represent the respective regions noted in column headers. The presence of a letter in a cell indicates significant differences between the indicated regions. Significance is at the 95% confidence interval.

**Table 3:** Treatment sequencing (n = 385)*

	Used first (n = 385), %	Used second (n = 166), %	Used third† (n = 39), %
ORS	41	10	8
Antibiotics	28	20	8
Anti-motilities	4	15	0
Zinc syrup	8	19	33
Herbal remedies	9	14	8
Zinc tablets	3	14	13
Other home remedies	1	1	18
Antibiotic injections	0	1	5
Other	7	7	6

ORS – oral rehydration solution

*Base: All respondents using treatment at last episode.

**†**Base too low for “used fourth” and for subgroup analysis.

Among ORS non–users, more than half used antibiotics, with especially high rates of use in the Nord and Centre regions (**Table 2**). Overall, most caregivers (57%) used only one treatment (ie, monotherapy), and only 10% used three or more treatments. Among caregivers who used only one treatment, 33% exclusively used ORS, and 30% exclusively used antibiotics. Among caregivers who did something to treat their child’s diarrhea (n = 385), 8% exclusively used a combination of ORS and zinc remedies and 5% used a combination of ORS, zinc, and other treatments.

Of the 177 children treated with ORS, 101 (57%) were less than 2 years old (**Table 4**). On average, caregivers who used ORS started doing so 1.8 days after the onset of diarrhea, administering it for an average of 2.8 days and using an average of 2.5 sachets during the episode. The average volume of ORS given per day to children less than 2 years old was approximately 600 ml, and children aged 2 to 5 years received an average of 740 ml.

**Table 4:** Dosing at last episode of diarrhea*

	Total (n = 177)	Children under 2 years (n = 101)	Children 2-5 years (n = 76)	Urban (n = 50) A	Rural (n = 127) B
Day started giving (mean)	1.8 (0.96)	1.8 (1.07)	1.8 (0.79)	1.8 (1.16)	1.7 (0.87)
For how many days (mean)	2.8 (1.39)	2.9 (1.24)	2.6 (1.56)	3.2 (1.81)	2.6 (1.13)
Number of sachets used during the episode (mean)	2.5 (1.06)	2.6 (1.01)	2.4 (1.12)	2.7 (1.16)	2.4 (1.01)
Amount given in one day (when the diarrhea episode was particularly bad; % children):					
250 ml	37	42	32	26, B	42
500 ml	12	13	11	14	11
750 ml	9	9	9	16, B	6
1000 ml	33	33	34	34	33
1250 ml	5	4	7	4	6
1500 ml	3	–	8	6	2

*Letters (A,B) represent the urban/rural regions as noted in column headers. The presence of a letter in a cell indicates significant differences between the indicated region. Significance is at the 95% confidence interval. Base: All respondents using ORS at last episode (n = 177). Standard deviation for means shown in brackets.

### Treatment goals and reasons for using treatments

Caregivers were asked to rank four key diarrhea treatment goals. They ranked “prevent child’s condition from getting worse” as the most important treatment goal, followed by “restore child’s energy and appetite.” “Reduce diarrhea motions” was the third most important, and “replace fluid lost due to diarrhea” was the lowest priority (**Table 5**).

**Table 5:** Caregiver ranking of key treatment goals (n = 400)*

	Most important (%)	Second most important (%)	Third most important (%)	Least important (%)
Prevent child’s condition from getting worse	81	13	2	4
Restore child’s energy and appetite	5	66	19	9
Reduce diarrhea motions	6	17	67	10
Replace fluid lost due to diarrhea	8	4	12	77

*Base: All caregivers (n = 400).

When caregivers who used ORS were asked why they decided to use this treatment, half said they were “instructed to do so by a nurse” (**Table 6**). Similarly, about half of those who used antibiotics reported that they had been given a prescription or a health care provider recommended it. Almost all those who used zinc products reported doing so because of a recommendation by a health care professional or community health worker.

**Table 6:** Reasons for using main treatments at last episode (% respondents)*

Reason	%
**Base: All caregivers using ORS at last episode (n = 177)**	%
Instructed to do so by nurse	50
Well known by caregivers and nurses	18
Recommended by someone	9
Rehydrate the child	7
Child to regain strength/energy	6
**Base: All caregivers using antibiotics at last episode (n = 144)**	
Medical prescription/ hospital or clinic recommendation	52
Treats diarrhea effectively	13
Treats diarrhea quickly	10
It kills the germ quickly at the start of diarrhea	8
**Base: All caregivers using zinc products (tablet and syrup) at last episode (n = 104)**	
Recommended by health care professionals/itinerant health agents	88
It is cheaper	12

### Caregiver perceptions of ORS

Overall, caregivers who were aware of ORS had positive perceptions of the product (**Table 7**). About two–thirds perceived ORS to be easy to prepare, and more than half said that clean water was easy to obtain and that instructions to prepare ORS were clear. About a third, however, reported that it was difficult to get the child to drink ORS, and many expressed uncertainty about whether use of ORS requires a young child to drink too much liquid or whether the frequency of giving ORS is acceptable.

**Table 7:** Caregiver perceptions (% response) of ORS, on positive–negative paired statements*

ORS–positive statement	Agree (%)	ORS–negative statement	Agree (%)	Don’t know (%)
Easy to prepare	66	Difficult to prepare	24	10
Reduces the child’s stooling	61	Does not reduce the child’s stooling	21	18
Not an expensive treatment	62	Expensive treatment	17	21
Increases child’s energy and appetite	57	Does not increase child’s energy & appetite	10	33
Is a medicine	70	Is not a medicine	14	16
Easy to obtain clean water to make it	58	Difficult to obtain clean water to make it	27	16
Stops the diarrhea	58	Does not stop the diarrhea	28	20
Easy to get the child to drink it	48	Difficult to get the child to drink it	32	20
Instructions on how to prepare it are clear	52	Instructions on how to prepare not clear	13	34
Not too much liquid for a young child to take	27	Too much liquid for a young child to take	30	43
Frequency of giving to the child is acceptable	27	Need to give to the child too often	27	46
Rarely have left over wasted liquid	20	Often have leftover waster liquid	50	30
Helps replace lost fluid/water & minerals	53	Does not help replace lost fluid/water & minerals	12	36
Stops vomiting	26	Does not stop vomiting	19	55
Necessary to treat diarrhea	12	Not necessary to treat diarrhea	63	24

ORS – oral rehydration solution

*Positive – chose ORS-positive statement; Negative – chose ORS-negative statement. Base: All caregivers who are aware of ORS (n = 328).

Most caregivers said they believed that ORS reduces the frequency of bowel movements, stops diarrhea, increases the child’s energy and appetite, and replaces lost fluid/water and minerals (**Table 7**). Nearly two–thirds of caregivers indicated that ORS is not an expensive treatment.

Caregiver perceptions of ORS varied substantially across regions (Table S3 in **Online Supplementary Document(Online Supplementary Document)**). For example, those in the Nord region especially perceived ORS as too much liquid for a young child to drink (56% agree).

Caregivers who did not use ORS at the last episode were prompted with a list of reasons for not using ORS and were asked to state whether the reason applied to them. The main reasons why ORS was not used pertained to administration and palatability: 24% said it was too difficult to get their child to take ORS, 24% said their child did not like the taste of ORS; and 20% reported having ORS left over that was then wasted (**Table 8**). Fewer than 10% of caregivers said that ORS was too difficult to obtain or too expensive.

**Table 8:** Caregiver reasons for not using ORS*

Caregiver reasons for not using ORS	%
It is too difficult to get my child to take ORS	24
My child does not like the taste of ORS	24
It was not recommended to me when I asked for advice	21
I often have ORS left over, which is wasted	20
You need to give ORS to the child too often	13
ORS is not effective at stopping diarrhea	11
It takes too long/is too far to travel to obtain ORS	10
It takes a lot of time and effort to make up ORS	7
Diarrhea is not a serious enough illness to justify using ORS	5
ORS is too expensive; other treatment options are cheaper	5

ORS – oral rehydration solution

*Base: All caregivers not using ORS at last episode (n = 208).

Asked to associate treatment types with a series of product attributes read aloud, ORS users associated ORS with positive attributes describing efficacy, availability, convenience, and trust (**Table 9**). Noticeably, ORS users, compared with non–users, more strongly associated ORS with being recommended by health care professionals, easily available, easy to prepare, good for restoring the child's energy and appetite, safe for children under 5 years old, and able to “significantly” reduce diarrhea motions. Additionally, ORS users were less likely to associate antibiotics with the same attributes, whereas non–users tended to associate both ORS and antibiotics with similar attributes.

**Table 9:** Comparative product associations*

	ORS sachet		Antibiotics		Anti-motility drugs		HSSS	
**Users (%)**	**Non–users (%)**	**Users (%)**	**Non–users (%)**	**Users (%)**	**Non–users (%)**	**Users (%)**	**Non–users (%)**
**A1**	**A2**	**B1**	**B2**	**C1**	**C2**	**D1**	**D2**
**Efficacy:**								
Helps replace fluid	56, A2	30	11, B2	29	4	5	11, D2	4
Safe for giving to under 5 year–old	62, A2	26	16, B2	33	5	8	11	6
Restores the child’s energy and appetite	66, A2	30	11, B2	31	3	6	7	6
Significantly reduced diarrhea motions	60, A2	21	19, B2	38	6	9	9	5
Stops the diarrhea	64, A2	29	21, B2	43	5, C2	11	10	6
Stops vomiting	35, A2	13	10, B2	22	2	4	7	4
**Availability:**								
Not expensive	72, A2	37	13, B2	40	3	6	13, D2	6
Easily available	73, A2	38	18, B2	43	4	8	11	8
**Convenience:**								
Easy to prepare	70, A2	33	18, B2	33	4, C2	10	10	7
Easy to take	55, A2	26	15, B2	31	3, C2	9	8	5
Nice tasting	44, A2	16	11, B2	24	2	6	9	6
Easy to use when traveling with a child	54, A2	27	17, B2	35	3, C2	11	4	3
**Trust:**								
Recommended by health care professionals	80, A2	44	23, B2	55	7	11	16	10

ORS – oral rehydration solutions, HSSS – homemade sugar and salt solution

*Letters (A1, A2, B1, B2, C1, C2, D1, D2) represent the respective user groups noted in column headers. The presence of a letter in a cell indicates significant differences between the indicated user groups. Significance is at the 95% confidence interval. Base: Users of ORS (n = 177); non-users of ORS (n = 208).

Among all caregivers, ORS was ranked most effective at treating diarrhea (40% vs 24% for antibiotics) and the best value for money (53% vs 16% for antibiotics) (Table S4 in **Online Supplementary Document(Online Supplementary Document)**). However, ORS non–users felt that antibiotics were more effective than ORS for treating diarrhea. Interestingly, ORS non–users said ORS and antibiotics had similar value for money. Also, there were substantial differences in rankings across regions.

When prompted to select a single preferred treatment (regardless of cost), caregivers generally preferred ORS. However, some regions showed a preference for antibiotics (Table S5 in **Online Supplementary Document(Online Supplementary Document)**). In addition, herbal remedies were especially popular in the Boucle du Mouhoun region, and zinc syrup was preferred by many in Hauts–Bassins.

### Treatment sourcing

Community health workers were the primary source of treatment recommendations for users of ORS or antibiotics (Table S6 in **Online Supplementary Document(Online Supplementary Document)**). Recommendations by doctors, nurses, or pharmacists were fairly infrequent. Most caregivers acquired ORS or antibiotics from pharmacists or public clinics/hospitals.

Additionally, 70% of caregivers said they had previously received information about ORS, and more than half of those in the Nord region (53%) and Est region (62%) recalled hearing about ORS within the past 3 months. Among the 282 caregivers who recalled hearing about ORS, information was most commonly heard at a health center (38%), at a hospital (22%), from neighbors or relations (20%), or from television/radio advertisements (18%).

### Access to health services

Among all caregivers, the average travel time to a pharmacy, a community health center, and a general public hospital was reported to be 23.2 minutes, 29.3 minutes, and 35.9 minutes, respectively (Table S7 in **Online Supplementary Document(Online Supplementary Document)**). The average travel time to a hospital varied across regions, from 25.5 minutes in Hauts–Bassins to 47.6 minutes in Nord.

Nearly half of all caregivers reported sometimes visiting a traditional healer when their child was ill (with any disease, not just with diarrhea) (Table S8 in **Online Supplementary Document(Online Supplementary Document)**). The greatest use of traditional healers occurred in the Est (81%) and Boucle du Mouhoun regions (59%).

### Financial considerations and preferred product formats

Nearly all caregivers who reported using ORS or antibiotics paid for the product (97% and 96%, respectively), with a median product cost of US$ 1.00 (CFA 1 = US$ 0.0020181 as of September 2014) for antibiotics and US$ 0.20 for a single ORS sachet (**Table 10**). In addition, nearly half (46%) of ORS users also purchased water to make the ORS.

**Table 10:** Total cost of ORS*

	Total (n = 177)	Centre (n = 21)	Boucle du Mouhoun (n = 46)	Hauts-Bassins (n = 45)	Nord (n = 32)	Est (n = 33)
Paid for product, %	96	95	100	93	94	97
Paid per sachet, median, US$	0.20 (0)	0.20 (0.18)	0.20 (0)	0.20 (0)	0.20 (0)	0.20 (0)
	**Total**	**Urban**	**Rural**			
All paying for ORS sachet	n = 170	n = 45	n = 125			
Median (total cost of sachets), US$	0.60 (0.40)	0.60 (0.60)	0.40 (0.20)			
All paying for water	n = 82	n = 26	n = 56			
Median (total cost of water), US$	0.60 (0.81)	1.00 (0.5)	0.60 (0.80)			
All paying for either sachet/water	n = 170	n = 45	n = 125			
Median (total cost of ORS + water), US$	0.80 (0.83)	1.10 (1.00)	0.60 (0.80)			

ORS – oral rehydration solution

*Base: All caregivers using ORS at last episode (n = 177). Interquartile range shown in brackets.

On average, caregivers spent a total of US$ 0.80 for both the ORS and the water. Rural caregivers tended to spend less (median = US$ 0.40 for ORS sachets and US$ 0.60 for water) than urban caregivers (median = US$ 0.60 for ORS sachets and US$ 1.00 for water). In addition to treatment costs, 51% of caregivers paid for other health–related expenses, such as consultation fees (92%; median = US$ 0.30) or a medical card fee (12%; median = US$ 0.30).

## CONCLUSIONS

Previous work has indicated that misperceptions among caregivers may be an obstacle to the adoption of ORS as the preferred treatment for diarrheal disease in children under five years of age in low–resource settings. We assessed caregiver knowledge and behaviors related to the treatment of affected children in Burkina Faso to inform strategic approaches for reducing morbidity and mortality. Specifically, we explored perceptions about ORS efficacy, alternative treatments, and obstacles to successful administration. Our conclusions do not address financing, policy, or manufacturing of ORS. These issues are well–discussed in previous research [7,8].

### Perception: ORS treats symptoms of diarrhea

Prior research has found that a primary contributor to nonuse of ORS in some countries is the perception that ORS is ineffective for treating the symptoms of diarrhea. This perception, often held by providers as well as caregivers, influences both whether ORS is prescribed/recommended and whether it is requested by caregivers [9]. A number of studies have shown that a sense of unmet expectations underlies this perception. Caregivers expecting ORS to reduce or stop the diarrhea may be disappointed by the product [10].

Our findings, however, suggest that the perception that ORS is ineffective may not be the largest barrier to uptake. Among ORS non–users in this study, only 11% mentioned lack of efficacy in stopping diarrhea as a reason for not using ORS. Our data suggest that most caregivers, in fact, believe that ORS stops diarrhea, and only a small proportion of caregivers (12%) ranked rehydration as the most important or second most important treatment goal. This suggests a fundamental lack of understanding of the critical contribution of dehydration to diarrheal–related deaths and the role of ORS in rehydration. Among ORS users, only 7% chose ORS because of its role in treating dehydration, whereas 68% reported using it because it was recommended by a nurse or was well known by caregivers and nurses. However, 56% of ORS users perceived that ORS helps to replace fluid compared to only 30% of non–users. These findings suggest that ORS usage is mostly due to recommendations by providers and not to an understanding of the core function of ORS. However, it does appear that more ORS users than non–users associate ORS with treating dehydration, suggesting that personal experience improves perception and understanding.

To set appropriate expectations among caregivers, communication messaging should focus on the seriousness of dehydration due to diarrhea and the ability of ORS to prevent dehydration. Our findings highlight the potential to increase uptake of ORS through clear, consistent communication around dehydration and the role of ORS. As suggested by Coreil and Genece, caregivers who understand the importance of ORS in treating dehydration are significantly more likely to use it [11]. Additionally, marketing ORS as a medicine to increase strength, rather than as an antidiarrheal, may improve usage given the concerns around unmet expectations as noted by Green et al. [12]. Surveyed caregivers in Burkina Faso identified increasing strength and energy as an important treatment goal, and ORS users already associated ORS with this goal.

Implications of our findings for future program development in Burkina Faso include a shift in messaging from use of ORS as an antidiarrheal to use of ORS to address dehydration and increase energy. Awareness–raising around the central role of dehydration in child mortality would be beneficial. Additionally, given the importance of recommendations by health workers, some communication/education efforts should focus on providers to increase their desire to recommend ORS.

### Perception: antibiotics and drugs offer a better alternative

Prior research has documented widespread, inappropriate use of antibiotics and other drugs for treatment of diarrhea. This is likely due to a preference by caregivers for treatments they see as “powerful” [13], especially for treating the symptoms of diarrhea. Because of the self–limiting nature of most cases of diarrhea, the point at which caregivers begin ORS administration, and the role of ORS in rehydration and not treating the symptoms, it is not surprising that caregivers seek a treatment that that they believe will give more immediate results in halting symptoms. In addition, there is a well–documented history of provider misinformation leading to recommendations of inappropriate treatments [14]. In a study in India, for example, 59% of prescriptions for children with diarrhea were for antibiotics [15].

Although we found ORS usage to be quite high in Burkina Faso (44% of caregivers used ORS at last episode), usage of antibiotics was also high (34%). Usage of both appears to be driven by professional recommendations, indicating a lack of consistent messaging among health care providers. ORS usage in Burkina Faso may be relatively high compared to antibiotic use, despite comparable views of efficacy, because ORS is perceived to be relatively inexpensive, easily available, and better value for money than antibiotics. Caregivers generally preferred ORS as their one preferred treatment, except in the Centre region, where antibiotics were preferred.

Zinc usage appears to be much higher in Burkina Faso than in India and Kenya [6]. Overall, 27% of caregivers used some form of zinc (either syrup or tablet) at the last episode of diarrhea. The relatively high use of zinc in Burkina Faso is likely due to the work of the Ministry of Health, supported by UNICEF and the Micronutrient Initiative. In a recent report, UNICEF noted that the proportion of community health centers using the combination of ORS and zinc to manage diarrhea went from 0% in 2010 to 42% by September 2012 [16]. Caregivers confirm this dramatic change, with 88% of those using zinc products noting a recommendation by a health care provider as the reason why they used the product.

The role of providers in decision–making and the fairly positive opinion of ORS efficacy among caregivers suggest that the elimination of inaccurate or mixed messaging regarding alternative treatments by providers will likely lead to improved use and outcomes. It is crucial for messaging to indicate that diarrhea will generally resolve without sophisticated or expensive medicines and that lost fluids must be replaced. Raising awareness about the rise of antibiotic resistance is a broader concern. Although pharmacist recommendation may be partly motivated by profit, providers are generally motivated to recommend the best available prescription despite caregiver requests for specific treatments. Well–informed providers and experienced mothers are the natural best advocates for successful dissemination of key messages.

### Perception: ORS is not user friendly

Prior research indicated that challenges with preparation and administration are the main reasons that ORS is perceived to not be user friendly. Measuring and mixing the proper amounts of ORS and water has been a challenge in household settings without proper measuring utensils [17]. In some settings, people describe duration and frequency of preparation and administration as a challenge [10]. Historically, adequate ORS dosing has been a hurdle, with most children consuming only minute quantities [18,19]. Palatability has also been investigated as an obstacle to administration of ORS. Findings from Burkina Faso echo findings from previous research studies in other countries in terms of the perception of ORS as not being user friendly.

Among caregivers aware of ORS, most do not have issues with administration aside from there being leftover liquid. However, 32% of caregivers in our study reported that it is difficult to get the child to drink ORS, and only 41% of children received a 1000 mL or more of ORS per day. Children under two and children in rural settings were at higher risk of receiving only a lower dose.

Our findings point to the potential for increasing ORS use through interventions to address ease of administration. The issue of leftover liquid may be addressed by offering a smaller ORS packet (200 mL, for example). Offering child–preferred flavors of ORS may be one way to improve upon the current difficulty of getting children to drink ORS. Unique product presentations such as a juice–box or pre–mix may provide one option to address administration and dosing challenges (though cost would need to be taken into consideration). In addition, a more sophisticated product presentation might simplify administration and enhance caregiver confidence that ORS is a “powerful” medicine. This is supported in the research by findings that the pre–mix was well received by caregivers. Flavor improvements might also improve uptake.

### Study limitations

Limitations of this study include:

Caregivers’ recall of reactions to a diarrhea event that happened up to 2 months before the interview may be inaccurate. Recall of measurements of treatments used may be particularly inaccurate. We tried to counter this by providing illustrations of bottles/cups and using the illustrations as an aid when asking respondents how much liquid was used.

There was some confusion among caregivers about which treatments fall into which categories. Caregivers often do not know what type of treatments providers advise/prescribe. Illustration cards with pictures of typical treatments that fall into each category (eg, antibiotics, anti–motility drugs, etc.) were used to aid recall.

Caregivers may have been influenced by illustration cards and just chosen a treatment shown that they didn’t actually use. However, we used spontaneous recall before using illustration cards, and the spontaneous and prompted recall of treatments was fairly consistent, indicating that prompted recall is reasonably reliable.

We did not probe into use of zinc (eg, doses used, timing, etc.). Higher–than–expected use indicates that motivations for use, sources of information, and usage practices, including use with ORS, should be explored further.
