# Supplementary material for: Caregiver perceptions and utilization of oral rehydration solution and other treatments for diarrhea among young children in Burkina Faso
Source: J Glob Health. 2016 Sep 16;6(2):020407. doi: 10.7189/jogh.06.020407 (PMC5032344; doi:10.7189/jogh.06.020407)
Supplement: Online Supplementary Document [file jogh-06-020407-s001.pdf]

Online Supplementary Document

Digre et al. Caregiver perceptions and utilization of oral rehydration solution and other treatments for diarrhea among young children in Burkina Faso

J Glob Health 2016;6:020407

Appendix S1. Supplementary tables.

Table S1. Regional demographics

Base: Dependent on region.							
		**Total (n=400)**	**Centre (n=90)**	**Boucle du Mouhoun (n=84)**	**Hauts-Bassins (n=86)**	**Nord (n=71)**	**Est (n=69)**
		%	%	%	%	%	%
Setting	Urban	38	100	13	35	13	10
	Rural	62	0	87	65	87	90
*Socioeconomic class**	C1	1	0	0	3	3	0
	C2	10	21	5	14	6	4
	D/E	88	79	95	83	92	96
Religion	Christian	30	44	23	12	21	54
	Muslim	66	56	68	86	77	42
	Other	4	0	9	2	1	4
Primary Language	French	22	42	21	22	11	10
	Moore	36	54	19	9	87	13
	Dioula	28	3	56	66	1	6
	Other	14	0	4	2	0	71

*Socioeconomic levels: A is highest and E lowest. Socioeconomic classification was based on the standard systems used for commercial market research in the respective countries; in Burkina Faso, as described in the Oracle General Consumer Survey – Brand Values Segmentation (GCS-S) data collection tool.

Table S2. Regions included in research.

Base: Dependent on region.						
	Caregiver sample			***Number of Sampling Points***		
	**Urban**	**Rural**	**Total**	**Urban**	**Rural**	**Total**
Centre	90	-	*90*	9	0	*9*
Boucle de Mouhoun	15	69	*84*	1	8	*9*
Hauts-Bassins	30	56	*86*	3	5	*8*
Nord	9	62	*71*	1	6	*7*
Est	7	62	*69*	1	6	*7*
TOTAL	151	249	*400*	15	25	*40*

Table S3. Caregiver perceptions (% agree response) of ORS by region.*

Base: All caregivers who are aware of ORS (n=328).						
	Total (n=328)	**Centre (n=71)**	**Boucle du Mouhoun (n=70)**	**Hauts-Bassins (n=70)**	**Nord (n=61)**	**Est (n=56)**
		**A**	**B**	**C**	**D**	**E**
Difficult to prepare	*24*	15	23	23	21	39
						A,B,C,D
Expensive treatment	*17*	6	13	11	30	29
					A,B,C	A,B,C
Is a medicine	*70*	87	66	60	66	73
		B,C,D,E				
Stops the diarrhea	*58*	66	63	57	41	62
		D	D			D
Instructions on how to prepare are clear	*52*	69	34	54	54	50
		B,E		B	B	
Too much liquid for a young child to take	*30*	38	13	11	56	36
		B,C			A,B,C,E	
Helps replace lost fluid/water and minerals	*53*	69	40	54	56	43
		B,E				
Stops vomiting	*26*	20	17	31	16	48
				D		A,B,D

* Letters (A,B,C,D,E) represent the respective regions noted in column headers. The presence of a letter in a cell indicates significant differences between the indicated regions. Significance is at the 95% confidence interval.

Table S4. Most effective and best value.

Base: All caregivers (n=400).	*Total (n=400)*	ORS users (n=177) A	ORS non-users (n=208) B	Centre (n=90) C	Boucle du Mouhoun (n=84) D	Hauts-Bassins (n=86) E	Nord (n=71) F	Est (n=69) G
Most effective at treating diarrhea	*%*	%	%	%	%	%	%	%
ORS	*40*	72 B	12	23	40 C	58 C,D,F,G	42 A	36
Antibiotics	*24*	7 B	38	31 D,E	6	9	45 D,E	30 D,E
Anti-motility drugs	*8*	3 B	13	21 D,E,F,G	6	5	0	9
HSSS	*3*	5	2	2	6	0	3	6
Best value for money	*%*	%	%	%	%	%	%	%
ORS	*53*	79 B	30	43	57	50	69 C,E,G	48
Antibiotics	*16*	3 B	29	29 D,E	1	8 D	27 D,E	19 D
Anti-motility drugs	*6*	5	7	10 E,F	6 E,F	0	0	12 E,F

* Letters (A,B,C,D,E, F, G) represent the respective regions noted in column headers. The presence of a letter in a cell indicates significant differences between the indicated regions. Significance is at the 95% confidence interval.

Table S5. Caregivers' one preferred treatment.*

Base: All caregivers (n=400).						
	Total (n=400)	**Centre (n=90)**	**Boucle du Mouhoun (n=84)**	**Hauts-Bassins (n=86)**	**Nord (n=71)**	**Est (n=69)**
		**A**	**B**	**C**	**D**	**E**
	%	%	%	%	%	%
ORS	*28*	18<br>B,D	32	24	37	29
Antibiotics	*19*	34<br>B,C,E	6<br>D,E	3<br>D,E	35<br>E	19
Zinc syrup	*11*	3<br>C,E	10<br>C	22	4	14
Anti-motility drugs	*7*	17<br>B,C,D	6	3	1	7
Herbal remedy	*7*	3<br>B	23<br>C,D,E	2	3	3
Zinc tablets	*3*	0	1	5	4	4
Another type of homemade remedy	*1*	1	1	0	0	1
Homemade salt and sugar solution	*0 †*	0	2	0	0	
Don't know/ can't say	*24*	23	19	40	15	22

* Letters (A,B,C,D,E) represent the respective regions noted in column headers. The presence of a letter in a cell indicates significant differences between the indicated regions. Significance is at the 95% confidence interval.

†Number too small for percentage on whole base.

Table S6. Treatment recommendations to caregivers and source of acquisition.

Base: All caregivers using at last episode.		
	**ORS (n=177)**	**Antibiotics (n=144)**
	%	%
I requested it	11	8
Who recommended?	n=158	n=133
Doctor	4	7
Pharmacist	12	5
Nurse	8	10
Community health worker	75	75
Other	1	3
Source of acquisition	n=177	n=144
Pharmacist	60	61
Public clinic/hospital	32	30
Private clinic/hospital	4	3
Had it at home	3	2
Other	1	3

Table S7. Average travel time (in minutes) to providers.*

	Total (n=400)	Centre (n=90)	Boucle du Mouchon (n=84)	Hauts-Bassins (n=86)	Nord (n=71)	Est (n=69)
		A	B	C	D	E
Pharmacy	23.2	18.4	28.2<br>A,C,D	22.6	21.4	26<br>A,D
Community health center	29.3	32.6<br>C	30.1	26	29.9	27.4
General public hospital	35.9	38.4<br>C	38.6<br>C,E	25.5	47.6<br>B,C,E	30.3

* Letters (A,B,C,D,E) represent the respective regions noted in column headers. The presence of a letter in a cell indicates significant differences between the indicated regions. Significance is at the 95% confidence interval.

Table S8. Reported visits to traditional healers.*

Base: All caregivers (n=400).						
	Total (n=400)	**Centre (n=90)**	**Boucle du Mouhoun (n=84)**	**Hauts-Bassins (n=86)**	**Nord (n=71)**	**Est (n=69)**
		**A**	**B**	**C**	**D**	**E**
Every time	*13*	7	20<br>A,C,D	9<br>D	1	30<br>A,C,D
Sometimes	*34*	22	39<br>A	26	34	51<br>A,C,D
Rarely	*20*	16	21	12	45<br>A,B,C,E	12
Never	*33*	56<br>B,D,E	19<br>E	53<br>B,D,E	20<br>E	7

* Letters (A,B,C,D,E) represent the respective regions noted in column headers. The presence of a letter in a cell indicates significant differences between the indicated regions. Significance is at the 95% confidence interval.

Appendix S2. Methods supplement.

The project team conducted a quantitative survey of 400 caregivers in Burkina Faso in June and July 2014. A complementary quantitative survey of 250 pharmacy staff and health care workers ("providers") was simultaneously conducted but is not covered in this report. Development of the survey instruments was informed by a formative qualitative research process, which involved 50 face-to-face, 60-minute, in-depth interviews with caregivers (predominantly mothers) who were aware of oral rehydration solution (ORS) as well as 50 interviewers with health providers. These qualitative interviews were conducted across five regions: Centre, Boucle de Mouhoun, Hauts-Bassin, Nord and Est.

Interviewer training

Prior to the quantitative data collection taking place, interviewers were extensively briefed about the research over a period of a week, and the interviewers had previous experience in conducting market research. This training took place in Ouagadougou in French. Among them, surveyors spoke the range of local languages (e.g., Moore, Dioula, Bambara, Fulfulde), as well as French. During this process interviewers were familiarized with the objectives of the research, the research materials and the expectations of conduct and approach whilst interviewing, as well as a reminder of ethical guidelines to adhere to. The interviewers also had a chance to ask any questions they had about the survey. Twenty pilot interviews were conducted as part of this training and a debriefing was conducted afterwards to address any challenges that were experienced during this testing of the survey. After this the final materials were refined and translated.

Selection and description of participants

All surveyed caregivers had a child between six months and five years of age who had an episode of diarrhea that occurred less than two months prior to the interview and that lasted for more than two days. Table 1 presents a demographic profile of caregivers. We used quota sampling methods to identify appropriate numbers of urban and rural respondents.

The survey covered five regions—Centre (including Ouagadougou, the capital), Boucle du Mouhoun, Hauts-Bassins, Nord, and Est—representing the country's major socio-cultural groups (see Table s1 for demographics of each region). Ensuring that rural communities would be appropriately represented in the data was a key consideration, as this is where the impact of poor health is often higher. However, it was also important to not neglect the practices and challenges of those living in urban settings. Therefore quota sampling was used based on the population distribution of Burkina Faso. The country was first of all stratified by the 13 provinces (regions) and 5 provinces were randomly selected (Table s2). Then within each province, the towns (urban and rural) were laid out. The selection of a principal town in each province had a probability of 1.00. The selection of other towns and rural locations within the province was random, with every rural location, however small, having a probability of being part of the sample. No town, urban or rural, was purposely excluded from the sampling process. Each of the selected study locations was stratified into high and low density sampling units. Within each of these sub-divisions, the sampling units were further divided into homogeneous sectors, to be randomly selected, from which the starting points were established. The starting point was where the interviewers would assemble to begin the random walk for the selection of dwelling structures, households, and finally the individual household member. The sampling gap used (interval of houses that would be approached to participate in the research) was every fifth house in high density areas and every third house in low density areas. If the household was not available when visited, the interviewer would go to the next household in the dwelling structure. However, only one substitution was allowed in any given dwelling structure. If the substitution failed again, then the interviewer would leave the entire dwelling structure and move to the next, where he would repeat the process of household selection.

Unlike the qualitative research, the quantitative research did not set criteria related to ORS awareness or experience so we could establish a representative measure of population awareness and usage of ORS and other diarrhea treatments.

Survey focus and design

The survey focused on the child's most recent episode of diarrhea. The two-month recall period allowed us to capture data in a reasonable amount of time and provided the possibility of more scope for a range of situations to be captured. This was piloted and the recall period proved appropriate as parents were able to think back to the last diarrhea episode in the two-month period and recall what happened. There were not many severe episodes (usually represented by longer disease duration) and average length of episode was a few days.

In the 60-minute interview, topics probed included diarrhea duration, treatments used, sequence/timeframe of administration, and caregivers' expectations for each treatment (e.g., "what did you think [the treatment] would do for your child?"). Caregivers were also asked about the treatment source, spending on treatment, care-seeking behaviors, and dosing of ORS and homemade sugar–salt solution (HSSS). The survey did not probe related costs such as transport or lost work time. Peak dosing estimates were calculated using conservative assumptions to err on the side of overestimating the amounts given. The survey questionnaire is available upon request from the corresponding author.

Other topics covered included awareness and previous use of treatments (ORS sachets, HSSS, other home remedies, herbal remedies, antibiotics, anti-motility drugs, and zinc syrups/tablets). After recording spontaneous recall of treatments used, interviewers used localized illustration cards to prompt or to assist in recall of treatment types. They also assessed perceptions of ORS by using positive–negative statement pairs and evaluated willingness to pay for a diarrhea treatment. Attribute association was carried out based on the four main treatments used (established previously in qualitative research): ORS, antibiotics, anti-motility drugs, and HSSS). This involved caregivers selecting which treatments they felt fulfilled each attribute (such as "easy for children to take," "stops the diarrhea," and "not expensive"). Interviewers also asked caregivers to rank the four treatments on effectiveness and value (HSSS was not included for the perception of value).

The survey was pretested with a small number of caregivers (n=20) in Ouagadougou. This ensured that survey questions were appropriate and refined before widespread data collection. A pen-to-paper approach was used for data collection.

Analysis

Data was systematically checked for inaccuracies and follow ups were conducted with respondents of surveys that did not make sense. Then data was transferred from the paper questionnaires to a computer data program (SPSS) and checked again to ensure that the correct number of people answered the appropriate questions. Data was analyzed for all respondents as a whole as well as for key groups, such as ORS users vs. ORS non–users (based on usage at last episode of diarrhea) and urban vs. rural respondents. We also evaluated data according to demographic and regional splits.

No clustering or weighting was used. Data was analyzed at an overall level and reviewed at sub-group level such as setting (urban/rural), ORS users vs. non-users, and region.

Data from open-ended questions were analyzed through a similar procedure. The process began with review of verbatim responses for each question. Key common themes were identified for each question, as well as factors associated with each theme. This represented a code frame. Each verbatim response was then analyzed and assigned to its appropriate code.
